# Analysis of the Level of Adiponectin and Selected Cytokines in Patients with Knee Osteoarthritis

**DOI:** 10.3390/medicina60040571

**Published:** 2024-03-30

**Authors:** Iosif Ilia, Paula Diana Ciordas, Diana Nitusca, Alina Anton, Catalin Marian

**Affiliations:** 1 Discipline of Biochemistry, “Victor Babes” University of Medicine and Pharmacy, 300041 Timișoara, Romania; iosif.ilia@umft.ro (I.I.); paula.muntean@umft.ro (P.D.C.); nitusca.diana@umft.ro (D.N.); cmarian@umft.ro (C.M.); 2 Faculty of Physical Education and Sport, Physical Activities Research Center, “Aurel Vlaicu” University of Arad, 310025 Arad, Romania; 3 Center for Complex Network Science, “Victor Babes” University of Medicine and Pharmacy, 300041 Timişoara, Romania; 4 Department of Toxicology and Drug Industry, Faculty of Pharmacy, “Victor Babes” University of Medicine and Pharmacy Timisoara, Eftimie Murgu Sq. No. 2, 300041 Timisoara, Romania; 5 Research Center for Pharmaco-Toxicological Evaluations, “Victor Babes” University of Medicine and Pharmacy Timisoara, Eftimie Murgu Sq. No. 2, 300041 Timisoara, Romania

**Keywords:** knee osteoarthritis, biomarkers, adiponectin, IL-6, IL-10, TNL-α

## Abstract

*Background and Objectives*: Knee osteoarthritis (KOA) is a degenerative disease that is continuously targeting people of different ages, but especially the elderly population, the number of which tends to increase continuously at the global level. Apart from age, excess weight can influence the evolution of the disease, with obesity being associated with a weak inflammation stage and an imbalance between pro-inflammatory and anti-inflammatory cytokines. The present work aimed to analyze specific biomarkers, namely ACRP-30, IL-10, TNF-α, and IL-6, in knee synovial fluid, and correlate them with KOA patients’ clinical data, radiographic changes, and functional and pain scores. *Materials and Methods*: 24 subjects with KOA and over 50 years of age participate in the present study. Synovial fluid was harvested using ultrasound guidance from the target knees of the enrolled KOA patients, and the levels of ACRP-30, IL-10, TNF-α, and IL-6 were measured using enzyme-linked immunosorbent assays (ELISA). All patients underwent a supine X-ray at the target knee and were classified using Kellgren–Lawrence (K–L) grading. The Western Ontario and McMaster University Osteoarthritis Index (WOMAC) was used to assess self-reported physical function, pain, and stiffness. *Results*: The obtained results highlighted a significant correlation between age and adiponectin level (*p* = 0.0451, r = −0.412). Also, the IL-10 values are lower in cases where the intensity of the pain is more pronounced (*p* = 0.0405, r = −0.421). In addition, analyzing the data by gender, it was observed that in the case of males, stiffness is more related to age (*p* = 0.0079, r = 0.7993), compared to women (*p* = 0.0203, r = 0.6223). In the case of women, the progression of the disease tends to increase more intensively the WOMAC score’s total values (*p* = 0.00031, r = 0.8342), compared with men (*p* = 0.0289, r = 7013). Regarding interleukins and BMI, significant correlations were observed only in the case of men. *Conclusions*: A significant correlation between age and adiponectin, and adiponectin and IL-6, suggests that advanced age may contribute to adiponectin reduction. Comparing men with women, it was observed that men’s age is more related to rigidity, and IL-6 and IL-10 are directly correlated to BMI; in addition, women seem to be more sensitive to pain and stiffness.

## 1. Introduction

Knee osteoarthritis (KOA) is a degenerative joint disease characterized by the gradual breakdown of cartilage in the knee joint. It is one of the most common types of arthritis, affecting millions worldwide [1]. The World Health Organization (WHO) assesses that by 2025 the number of the elderly population will grow by 414% compared to the year 1990 [2]. Moreover, estimates suggest that the proportion of people aged 65 and above will reach 16% of the worldwide population [3]. Thus, this phenomenon leads to an increase in cases of prevalence of KOA [1]. A study confirmed that KOA in American adults is responsible for 80% of the total osteoarthritis cases and affects about 20% of those over 45 years old [4]. Another study from the United States (US) estimated the incidence of symptomatic KOA with the use of self-reported population-based data, showing that approximately 9.29% of subjects older than 60 years have been already diagnosed with symptomatic KOA [5]. This disease has an important impact on affected persons’ quality of life; the progressive and chronic condition and high prevalence also affect socio-economic status. 

**Figure 1:**
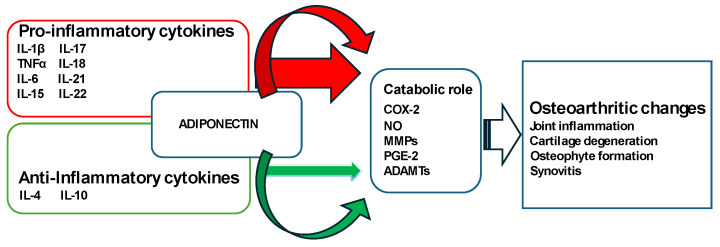
Pathogenesis of osteoarthritis: schematic representation of the disturbed balance of pro-inflammatory and anti-inflammatory cytokines (in favor of pro-inflammatory cytokines) responsible for the secretion of enzymes and other inflammatory factors with catabolic roles leading to structural changes within the joint such as cartilage degeneration, osteophyte formation, and synovitis.

Besides old age, obesity can be a trigger or aggravating factor in the development of KOA [6]. Excess weight puts added stress on the knee joints, accelerating cartilage degeneration. Obesity is associated with a weak inflammation stage, and the deterioration of cartilage may be attributed to an imbalance between pro-inflammatory and anti-inflammatory cytokines, leading to destructive effects [7]. Adipose tissue plays a crucial role in the development and progression of KOA through metabolic, biomechanical, and pro-inflammatory factors that trigger illness. It has been recognized as a powerful internal endocrine organ due to its ability to release biologically active adipokines, such as adiponectin (ACRP-30), a 244-amino acid polypeptide that participates in various physiological and pathological processes. The expression of ACRP-30 in serum and adipose tissue is reduced in individuals with obesity [8]. It represents the majority of adipokines from circulation, being structurally homologous to tumor necrosis factor-α (TNF-α) [9]. It has been observed that ACRP-30 manifests the anti-inflammatory potential by downregulating cytokines with pro-inflammatory properties, e.g., TNF-α and interleukin-6 (IL-6), and by stimulating the expression of cytokines with anti-inflammatory attributes [10]. TNF-α seems to have a crucial function in the breakdown of cartilage matrix and KOA. It has the ability to stimulate the generation of matrix metalloproteinases, IL-6, and prostaglandins, while also downregulating the production of type II collagen and proteoglycans [11]. In a Dutch study, it was observed that the ex vivo level of TNF-α from whole blood samples stimulated with lipopolysaccharide was related to the radiological progression of KOA over two years [12]. IL-10 is another important biomarker, being able to interfere with TNF-α production and other mediated events correlated with osteoarthritis development [13], hence indicating the significance of a controlled equilibrium between anti-inflammatory and pro-inflammatory cytokines in maintaining the health of the joints. KOA progression is known to be linked to an elevated production of TNF-α and IL-10 in whole blood, where findings suggest that concentrations of local and circulating cytokines decrease in advanced KOA compared to moderate cases [14]. A schematic representation of the pro- and anti-inflammatory cytokines and other molecules involved in the KOA pathogenesis is depicted in Figure 1 [15].

The early identification of patients at risk of developing KOA and the anticipation or correction of predisposing factors are essential in disease management. Diagnosis is usually based on the patient’s history and clinical features, according to the American College of Rheumatology criteria for the diagnosis of KOA [16]. Nevertheless, in several cases, particularly in patients displaying suspected clinical characteristics, the verification of KOA or the assessment of joint involvement may necessitate the execution of radiography or magnetic resonance imaging (MRI) scans [17]. Systemic biomarkers of KOA have extensively been searched, especially in urine and serum samples; however, their concentrations could be more prone to systemic influences, which can interfere with diagnosis. Furthermore, KOA is characterized by the degradation of cartilage and other local tissues involved; thus, synovial fluid (SF) can reflect more closely the alterations caused by KOA in a singular joint and seems to be suitable for studies due to its direct contact with the affected cartilage.

Led by the plethora of addressed evidence, the aim of the present study was to analyze specific biomarkers like ACRP-30, IL10, TNF-α, and IL-6 in SF and correlate them with KOA patients’ clinical data, radiographic changes, and patient-reported outcome measures (PROMs). 

## 2. Materials and Methods

### 2.1. Patient Selection

The group involved in the present study consists of 24 patients over 50 years of age, with knee pain and joint effusion detected both clinically and by ultrasound, diagnosed with KOA in our Outpatient Physical Rehabilitation Center, in Timisoara, Romania, between January 2022 and September 2023. Every patient who was included in the study group homologized with the inclusion criteria, signed a consent agreement form, and did not carry out any of the exclusion criteria. The present study was guided according to the Declaration of Helsinki and the design was approved by the Local Ethical Committee at the “Victor Babes” Medical University of Timisoara, Romania (75/10 December 2021). 

The inclusion criteria for subjects were as follows:

1. Written consent for participation signed;
2. Accomplishing the criteria of the KOA diagnosis according to the American College of Rheumatology: pain at the level of knee + at least one of the following conditions:
  - Age > 50 years;
  - Stiffness < 30 min;
  - Crepitus on active motion—plus osteophytes.
3. Existence of the joint effusion identified and aspirated by ultrasound guidance;
4. None of the exclusion criteria.

The exclusion criteria for subjects were as follows:

- Age < 50 years at diagnosis;
- Previous surgical intervention on the target knee;
- Anterior injections with steroids or hyaluronans to the target knee (in the last 6 months);
- The presence of knee infections or an infection within 3 months before enrollment;
- History of knee trauma (in the last 6 months).

### 2.2. Clinical Evaluation

Demographic data were collected, including gender and age, height and weight were measured, and body mass index (BMI) was calculated; the presence of knee pain, crepitus, joint stiffness, and clinical joint effusion were noted. Pain, stiffness, and physical function were evaluated using the Western Ontario and McMaster Universities Osteoarthritis Index (WOMAC), composed of three subscales, including pain (five items, 0–20 points), joint stiffness (two items, 0–8 points), and physical function (17 items, 0–68 points). The final score for the WOMAC was determined by adding the aggregate scores for pain, stiffness, and physical function. Scores range from 0 to 96 for the total WOMAC, where 0 represents the best health status and 96 the worst possible status. The higher the score, the poorer the function [18]. Pain assessment in these patients includes the use of a generic unidimensional visual analogue scale (VAS), also.

Body mass index (BMI) was calculated on the day of the joint fluid collection according to the following formula:

BMI = weight (kg)/height (m)^2^. (1)

If someone’s BMI was between the 25–29.9 range in the BMI chart, they were considered overweight; if their BMI went above 30, they fell within obesity territory. Table 1 presents a BMI chart, according to the World Health Organization’s (WHO) BMI guidelines. 

### 2.3. Imaging

All patients performed a supine X-ray, where the target knee was scanned from both antero-posterior and lateral views. Then, the stage of KOA was assessed according to the Kellgren–Lawrence (K–L) scale [7]. The K–L scale uses five grades (none, doubtful, minimal, moderate, and severe) to classify the severity of KOA and is one of the most used classification methods. Its explanation is as follows:

- None/grade 0 = definite absence of X-ray changes in osteoarthritis;
- Doubtful/grade 1 = uncertain joint space narrowing, uncertain osteophytic lipping;
- Minimal/grade 2 = definite joint space narrowing and osteophytes;
- Moderate/grade 3 = moderate multiple osteophytes, definite narrowing of joint space, sclerosis, and eventual deformity of bone ends;
- Severe/grade 4 = large osteophytes, significant narrowing of joint space, marked sclerosis and obvious deformation of bone ends.

Ultrasound was performed using an ESAOTE MyLab Omega (ESAOTE Spa, Genova, Italy) ultrasound device and a 6–19 MHz linear array transducer immediately after the clinical evaluation to confirm the joint effusion as well as to guide the fluid aspiration.

The same physician achieved all evaluations, to avoid intrasession variability. 

### 2.4. Obtaining and Evaluation of the Material

The specimen of SF was obtained from the suprapatellar recess by ultrasound-guided aspiration. The volume was then aliquoted, frozen, and stored at −80 °C for further analysis. SF samples were defrosted on the day of testing, one aliquot for a single test, and evaluation of IL-10, IL-6, and TNF-α concentrations was performed using commercial ELISA kits: Invitrogen IL10 Human ELISA Kit EHIL10 (Thermo Fisher Scientific Inc., Waltham, MA, USA), Invitrogen IL6 Human ELISA Kit EH2IL6 (Thermo Fisher Scientific Inc., Waltham, MA, USA), and Invitrogen TNF alpha Human ELISA Kit KHC3011 (Thermo Fisher Scientific Inc., Waltham, MA, USA). To assess the concentration of adiponectin, the following ELISA kit was used: Adiponectin (total) ELISA Kit (Immunodiagnostik AG, Bensheim, Germany). 

The protocols were respected according to manufacturers’ Instructions and the measurements were performed at the Department of Biochemistry, “Victor Babes” Medical University of Timisoara, Romania, using a GloMax^®^ Discover Microplate Reader (Promega Inc., Fitchburg, MA, USA). All experiments were performed in duplicate, and the mean value of two separate measurements was used as a result.

### 2.5. Statistical Analysis

Statistical analyses were performed using the GraphPad Prism version 6.0.0 software for Windows (GraphPad Software, La Jolla, CA, USA). The D’Agostino and Pearson test was applied to analyze the data. Correlations were performed with the two-tailed Spearman test. All the presented experimental data are as means ± standard deviation (SD). The obtained statistically significant differences between results were highlighted with * (* *p* < 0.1; ** *p* < 0.01; *** *p* < 0.001; **** *p* < 0.0001). The evaluation of the correlation’s strength was conducted using the subsequent points of reference: r = 1—full correlation, 0.9 < r < 1.0—almost complete correlation, 0.7 < r ≤ 0.9—very strong correlation, 0.5 < r ≤ 0.7—strong correlation, 0.3 < r ≤ 0.5—medium correlation, 1 < r ≤ 0.3—weak correlation, 0.0 < r ≤ 0.1—very weak correlation, and r = 0—no correlation.

## 3. Results

The studied group consisted of 24 patients respecting the inclusion criteria, with a close distribution of both sexes, 10 males (41.7%) and 14 females (58.3%). The demographic and clinical characteristics are presented in Table 2. The mean age was 67 years, and the BMI indicates that most of the subjects involved were overweight. The calculated K–L score shows that the majority of patients present grade 2/3 KOA, with possible joint space narrowing, moderate multiple osteophytes, sclerosis, and eventual deformity of bone ends. In addition, most subjects manifest moderate pain, but significant rigidity. The physical function score highlights that the activities of daily living (ADLs) are moderately affected. The WOMAC pain subscale and the visual analogue scale (VAS) suggest similar moderate pain and discomfort in most cases.

The mean values of ACRP-30 (ng/mL), IL-6 (pg/µL), IL-10 (ng/µL), and TNF-α (pg/mL) levels presented in SF are presented in Table 3. 

When comparing demographic and clinical characteristics of the studied group with biomarkers found in patients’ SF (Table 4), negative correlations were found between age and ACRP-30 (*p* = 0.0451, r = −0.412), advancing in age being associated with a reduction in adiponectin levels. Also, the IL-10 values are lower in cases where the intensity of the pain is more pronounced (*p* = 0.041, r = −0.421). In addition, BMI was associated with the reduction of both tested interleukins (*p* = 0.168 for IL-6, and *p* = 0.104 for IL-10). Stiffness was related to IL-10 reduction (*p* = 0.15, r = −0.302). 

When analyzing the interdependence of the monitored biomarkers, positive correlations were obtained between ACRP-30 and IL-6, adiponectin levels being associated with IL-6 augmentation levels (*p* = 0.045, r = 0.413). The increased values of IL-6 were also associated with the significant increase in IL-10 values (*p* = 3.42 × 10^−7^, r = 0.837) (Table 5).

The next step of the study was to stratify the patients by gender, in order to identify some characteristics specific for men/women. Data correlations are depicted in Table 6 and Table 7. In the case of both sexes, age is positively correlated to the WOMAC pain subscale, WOMAC stiffness subscale, WOMAC ADL and total WOMAC scores. Pain is also associated with age in both cases (*p* = 0.0439 in the case of men and 0.0417 in the case of women). Though in the case of males, stiffness is more related to age (*p* = 0.0079, r = 0.7993), compared to women (*p* = 0.0203, r = 0.6223). The KOA severity is positively correlated to the WOMAC pain subscale score, the stiffness, ADL, VAS scores, and the total WOMAC score; however, according to the K–L score, the progression of the disease tends to correlate more intensively with the total WOMAC score’s values in the case of women (*p* = 0.0003, r = 0.8342) (Table 7), compared with men (*p* = 0.0289, r = 7013) (Table 6). The WOMAC pain subscale score is also positively correlated to stiffness, ADL, and the total WOMAC scores, which are more pronounced in the case of women (*p* < 0.0001 in all the cases), compared to men (*p* = 0.0094, 0.0057, and respectively 0.0030). Regarding interleukins and BMI, significant correlations were observed only in the case of men (*p* = 0.0239 for IL-10 and 0.0234 for IL-6). 

## 4. Discussion

KOA remains a significant health problem and identification of risks for incidence or disease progression remains challenging, as radiography, the most common method of identification, is not sensitive enough to mirror the molecular changes that can occur in affected cartilage and bone [20]. The aim of the present work was to correlate the patients’ clinical data with specific biomarkers like ACRP-30, IL-10, TNF-α, and IL-6 found in SF.

By analyzing demographic data, in the present study it was observed that women had a slightly higher prevalence compared to men (58.3% vs. 41.7%). A moderately higher prevalence among women was also observed in several studies, thus a recent review including 88 studies with 10,081,952 subjects observed that the proportion of prevalence and incidence in females/males were 1.69 (95% CI, *p* < 0⋅00) and 1.39 (95% CI, *p* < 0⋅00), respectively [21]. It was also noticed in our study that the majority of subjects with KOA were overweight. Previous research studies investigated whether age and BMI can facilitate the identification of accelerated KOA compared with the common form of the illness. They concluded that older people with higher BMI and other injuries were more liable to develop accelerated KOA than the common form [22].

One of the most common methods of classification of KOA is the use of the K–L scale [23,24]. In this case, results highlighted that two/three were the most encountered grades, bringing out that patients with joint effusion present minimal to moderate scores of KOA. However, these patients complain of moderate pain, but more pronounced stiffness, which affects ADL. Thus, in our case, pain and discomfort seem not to be the major cause of alarm for patients, but rather the affectation of joint elasticity. A recent study confirmed that the incidence of pain and tenderness in the case of KOA is 36.8–60.7%, this being most often accompanied by limited activity, joint deformities, bone rub feeling, and muscle atrophy [25]. 

Biomarkers serve as essential tools for predicting disease and distinguishing between pathological and physiological events. Essentially, biomarkers can provide a pathway for studying the early progression of KOA. ACRP-30 is secreted by white adipose tissue and is usually found in SF and cartilage tissues of patients with KOA [26,27]. The role of adipokine in KOA remains indefinite. Certain researchers discovered a protective effect of ACRP-30 in KOA [10,28], while others observed no correlation [29], or on the contrary, a positive correlation, ACRP-30 being associated with both the radiological and clinical severity of KOA [30]. In our study, a new association was found, between age and ACRP-30, advancing age being correlated with the reduction in ACRP-30 levels. However, when analyzed separately by gender, a weaker correlation was observed in the case of both sexes, with the male sex having a *p*-value of 0.0643 (r = −0.6127) and the female sex having a *p*-value of 0.2856 (r = −0.3068). The lack of statistical significance in this case could be due to data stratification, thus reducing the number of subjects in each group, the limited number of included subjects being a clear limitation of this study. In addition, adiponectin levels were positively associated with increased IL-6 levels. A similar effect was observed in another study, where the association of pro-inflammatory agents with ACRP-30 was analyzed in the SF of arthritis patients. Results showed that ACRP-30 functioned synergistically with IL-1β to activate IL-8 and IL-6 expression in synoviocytes [31]. Another study showed that in cultured chondrocytes, ACRP-30 treatment increased, in a dose-dependent manner, the pro-inflammatory factors such as IL-6, inducible nitric oxide synthase, and metalloproteases [32]. Contrarily, Udomsinprasert et al. observed that ACRP-30 levels from SF were negatively associated with C-reactive proteins and IL-6 levels and positively correlated with 25(OH)D levels in KOA-affected patients [33]. In another study, in osteoarthritis patients, there were significant positive correlations between ACRP-30 levels in the synovial membrane and plasma levels of IL-4, IL-1β, G-CSF, and GM-CSF. Contrarily, in patients with rheumatoid arthritis, there were no observed significant correlations between adiponectin levels and plasma levels of cytokines [34]. Moreover, another study reported that elevated ACRP-30 levels promote IL-8 and IL-6 secretion in rheumatoid arthritis patients, suggesting that augmented adiponectin levels promote the illness [35].

Pro-inflammatory cytokines including IL-6 and TNF-α have been demonstrated to present catabolic effects by reducing collagen synthesis or increasing matrix metalloproteinases [36]. Instead, immunoregulatory and anti-inflammatory cytokines, such as IL-10, suppress pro-inflammatory expression and stimulate cartilage synthesis [37]. A disequilibrium in the anabolic and catabolic processes in affected joints results in the degradation and depletion of cartilage, processes that may be directly affected by cytokine imbalance.

Furthermore, the increased values of IL-6 were also associated with the significant increase of IL-10 values (Table 5). IL-10 is a cytokine with significant anti-inflammatory potential that broadly has to downregulate pro-inflammatory cytokine activity. Similar to our findings, Mabey and colleagues observed that median plasma levels of pro-inflammatory cytokines (IL-6) and anti-inflammatory ones (IL-2, IL-4, IL-10) were significantly superior in patients with KOA compared to healthy subjects (controls) [36]. A significant upregulation of IL-10 was discovered in patients subjected to physical exercises, compared to sedentary ones. In one study, in the exercise group, over the 3 h post-physical effort, IL-10 remained augmented, while IL-10 remained unchanged over time in the non-exercise group. Instead, IL-6 showed significant increases over time in both groups [38]. Also, the IL-10 values were found to be lower in cases where the intensity of the pain and stiffness were more pronounced. A reduction in interleukins was also associated with BMI. Thus, the heavier the patients, the lower the amount of cytokines was observed to be (*p* = 0.168 for IL-6 and *p* = 0.104 for IL-10). 

When the data were analyzed stratified by sex, according to Table 6 and Table 7, pro-inflammatory cytokines positively correlated with the K–L score. However, they negatively correlate with age, BMI, and the WOMAC subscales in the case of men. Similar findings were observed in the study by Veličković et al., where TNF-α was negatively correlated with the WOMAC pain and stiffness subscales. IL-6 was positively correlated with the WOMAC subscales but without significant associations [39]. Instead, in the case of women, age, BMI, and the WOMAC pain, stiffness, and physical function subscales are positively correlated with IL-6, however, without significant association. TNF-α is positively associated with BMI, the WOMAC pain and ADL subscales, the total WOMAC, and the VAS score. Nevertheless, there are no significant correlations. As in the case of this study, in the research conducted by Li et al., no significant correlation was observed between the WOMAC score and pro-inflammatory mediators, but it was associated with a range of motion assessments. In addition, IL-6 levels increased with advanced stages. The numeric rating scale was associated positively with the WOMAC scores and the K–L grade, and negatively with the range of motion and TNF-α [40].

In addition, it was observed that in the case of males, rigidity is more related to age (*p* = 0.0079), compared to women (*p* = 0.0203). Moreover, in men, a significant relationship was observed between BMI and interleukins. The disease tends to increase more intensively the total WOMAC values (*p* = 0.0003) in women compared with men (*p* = 0.0289). This observation could be related to the fact that women are more sensitive than men, in the case of KOA manifestations, especially in the case of pain [41]. These intersexual differences have attracted the attention of sex-specific therapeutic approaches and responses. The main treatment goal for KOA is to improve the quality of life, ameliorate motor function, prevent disability, and reduce pain [42]. A recent study observed that in postmenopausal women, physical effort has been positively associated with amelioration in coordination, balance, flexibility, muscle strength, and bone mineral density [41]. In addition, de Marziani et al. observed that women showed a greater temperature decrease than men when they were evaluated with a thermographic camera, directly after and five minutes after a 2 min knee flexion–extension exercise [43]. Another study aimed to evaluate the impact of sex on the effectiveness of platelet-rich plasma (PRP) in KOA patients. The PRP did not contain leucocytes or erythrocytes and had a platelet concentration factor of 2.0 × compared to blood levels. The knee injury and osteoarthritis outcome scores highlighted a significant increase from the baseline to six months, with augmentation in the physical component summary and mental component summary. Compared with men, the number of knees of women with minimal clinically important improvement was higher. In addition, women presented worse baseline scores in the mental component summary and pain [44].

The WOMAC pain subscale score is also positively correlated to stiffness, ADL, and total WOMAC scores, which are more pronounced in the case of women. These data suggest that women with KOA are more sensitive to pain and articular rigidity. Another study also pointed out that females have significantly greater pain sensitivity, more pain, more impairment on specific functional tasks, and poorer perceived function [45]. The anatomical variances between females and males could potentially have an impact in the evolution of the disease, but also on the symptomatic manifestations, consisting of thinner patellae, narrower femurs, larger quadriceps angles, and variations in tibial condylar size [46]. Besides these anatomical differences, kinematic differences like greater anterior and posterior shear forces, greater valgus moment, and extension, may also play a role in the development of KOA in women [47]. The increased OA frequency in postmenopausal women and the increased risk of developing arthritis led to the hypothesis that female hormones, especially the decrease in estrogen, play a causative role [48]. This hypothesis is sustained by Zhang who demonstrated that estrogen replacement therapy may decrease the chance of developing radiographic KOA [49]. Women have demonstrated lower functional scores before total knee arthroplasty [50] and more advanced disease at the time of surgery, which translated into increased pain, muscle atrophy, and impaired quadriceps function [51].

General and pain-related psychological distress, such as fear avoidance beliefs, catastrophizing, low self-efficacy, work-related stress, depression, and/or anxiety, can negatively influence disability, quality of life, and treatment outcomes for individuals with KOA, and is more pronounced in women; thus, identifying and addressing these yellow flags would be essential for a better understanding of PROMs data, in preventing chronic pain, for providing more comprehensive and tailored care in these patients, and for promoting motivation in returning to their daily activities.

The study has some clear limitations, mainly the lack of a control group of healthy subjects, and the rather small sample size. Consequently, the results obtained in the course of the analyses should be treated as preliminary, and subsequent studies with a larger number of participants are warranted to confirm the statistical trends and dependencies that have been observed.

The collection and assessment of SF has had several drawbacks as well. Although it covers the intrinsic structures of the diarthrodial joint, providing a rare chance to assess the entire joint, it was more challenging to harvest in clinical practice despite the use of ultrasound guidance, and its evaluation was occasionally constrained by the small volume. For ethical reasons, we did not intend to obtain any synovial fluid from the healthy knee or the asymptomatic contralateral to use as a comparison. There was no blood drawn in these patients either. The drawback of this approach is that we cannot determine whether the obtained biomarker values are condition-specific, related to the target joint, or if they are subject to systemic influences.

## 5. Conclusions

Knee osteoarthritis remains a challenging problem affecting millions of people daily. Biomarkers for this disease are continuously researched. In the present study, IL-6, TNF-α, IL-10, and adiponectin were studied. The results highlighted a significant correlation between age and ACRP-30, and ACRP-30 and IL-6, suggesting that advanced age may contribute to adiponectin reduction, and this also manifests a synergistic effect with IL-6. At the same time, IL-6 increase attracts IL-10 augmentation. Comparing men with women, it was observed that men’s age is more related to rigidity, and IL-6 and IL-10 are directly correlated to BMI. Rather, women seem to be more sensitive to pain and stiffness. 

Understanding how these biomarkers work would be essential in an early diagnosis, predicting the disease, or a rapid progression of the disease, and in developing new disease-modifying osteoarthritis drugs (DMOADs), not only to relieve the clinical symptoms but also to slow down joint space narrowing on X-rays.

## Figures and Tables

**Table 1:** BMI chart per the WHO BMI guidelines [19].

BMI	Weight Status
<18.5	underweight
18.5–24.9	healthy weight
25–29.9	overweight
≥30	obesity

**Table 2:** Demographic and clinical characteristics of the studied group.

Variable	Mean ± SD or *n* (%)	Minimum	Maximum
Number of patients	24		
Gender (males/females)	10(41.7%)/14(58.3%)		
Age (years)	67 ± 10.08	51	87
BMI	28.21 ± 3.47	24	35
Kellgren–Lawrence score	2.292 ± 1.16	0	4
WOMAC pain subscale	5.625 ± 2.7	2	12
WOMAC stiffness subscale	1.917 ± 1.06	0	3
WOMAC physical function subscale	20.21 ± 6.77	9	33
Total WOMAC score	28.04 ± 10.2	12	47
VAS score	5.83 ± 1.71	3	9

**Table 3:** Concentrations of the studied biomarkers in synovial fluid.

Variable	Mean ± SD or *n* (%)	Minimum	Maximum
ACRP-30	3420 ± 1219	1046	6943
IL-6	1518 ± 762.4	11.68	18,364
IL-10	6.327 ± 10.83	0.1	50.18
TNF-α	7.15 ± 3.24	3.52	17.48

**Table 4:** Correlation of demographic and clinical characteristics with biomarkers.

Variable	Age (Years)	BMI	Kellgren–Lawrence Score	WOMAC Scale				VAS Score
				Pain	Stiffness	ADL Score	WOMAC Total	
ACRP-30	−0.412	−0.056	−0.122	−0.216	−0.13	−0.025	−0.093	−0.09
	0.045 *	0.792	0.567	0.309	0.542	0.904	0.663	0.675
IL-6	0.023	−0.29	0.127	−0.24	−0.116	0.001	−0.095	−0.067
	0.912	0.168	0.553	0.258	0.588	0.993	0.658	0.752
IL-10	−0.457	−0.339	−0.105	−0.421	−0.302	−0.198	−0.247	−0.213
	0.832	0.104	0.622	0.041 *	0.15	0.374	0.242	0.317
TNF-α	0.04	−0.038	0.193	−0.06	−0.023	0.11	0.054	0.148
	0.85	0.857	0.365	0.779	0.913	0.605	0.8	0.488

Statistically significant values are marked with *, as follows: * *p* < 0.1.

**Table 5:** Correlation of biomarkers in synovial fluid.

Variable	ACRP-30	IL-6	IL-10	TNF-α
**ACRP-30**				
			
**IL-6**	0.413			
	0.045 *			
**IL-10**	0.297	0.837		
	0.158	<0.001 ****		
**TNF-** **α**	0.181	0.229	0.249	
	0.398	0.281	0.24	

Statistically significant values are marked with *, as follows: * *p* < 0.1; **** *p* < 0.0001.

**Table 6:** Correlation data of demographics, clinical characteristics, and biomarkers found in men.

	Age	BMI	Kellgren–Lawrence Score	WOMAC Pain Subscale	WOMAC Stiffness Subscale	WOMAC Physical Function	Total WOMAC Score	VAS Score	IL-10	IL-6	ACRP-30	TNF-α
Age		0.2876	0.5614	0.6543	0.7993	0.7675	0.7478	0.5144	−0.4610	−0.4377	−0.6128	−0.2376
		0.4177	0.0956 *	0.0439 *	0.0079 **	0.0109 *	0.0146 *	0.1330	0.1820	0.2080	0.0643 *	0.5065
BMI	0.2876		0.2032	0.3537	0.4557	0.2439	0.1101	0.2168	−0.7173	−0.7212	−0.4667	0.0061
	0.4177		0.5790	0.3135	0.1857	0.4945	0.7628	0.5449	0.0240 *	0.0234 *	0.1786	1.0000
Kellgren–Lawrence score	0.5614	0.2032		0.6926	0.6831	0.8245	0.7014	0.8978	−0.1381	0.0590	−0.3999	0.2688
	0.0956 *	0.5790		0.0317 *	0.0290 *	0.0052 **	0.0290 *	0.0008 ***	0.7101	0.8849	0.2575	0.4599
WOMAC pain subscale	0.6543	0.3537	0.6926		0.8024	0.8160	0.8492	0.8755	−0.5382	−0.4512	−0.5671	−0.2622
	0.0439 *	0.3135	0.0317 *		0.0094 **	0.0057 **	0.0030 **	0.0015 **	0.1117	0.1916	0.0923 *	0.4613
WOMAC stiffness subscale	0.7993	0.4557	0.6831	0.8024		0.9366	0.8508	0.6886	−0.6203	−0.4492	−0.4101	−0.1237
	0.0079 **	0.1857	0.0290 *	0.0094 **		0.0001 ***	0.0034 **	0.0337 *	0.0614 *	0.1929	0.2392	0.7320
WOMAC physical function	0.7675	0.2439	0.8245	0.8160	0.9366		0.9415	0.8163	−0.3914	−0.2378	−0.3476	0.0000
	0.0109 *	0.4945	0.0052 **	0.0057 **	0.0001 ***		0.0001 ***	0.0056 **	0.2599	0.5056	0.3231	1.0000
Total WOMAC score	0.7478	0.1101	0.7014	0.8492	0.8508	0.9415		0.8001	−0.3865	−0.3058	−0.3853	−0.2080
	0.0146 *	0.7628	0.0290 *	0.0030 **	0.0034 **	0.0001 ***		0.0078 **	0.2663	0.3870	0.2702	0.5619
VAS score	0.5144	0.2168	0.8978	0.8755	0.6886	0.8163	0.8001		−0.2454	−0.1301	−0.5326	0.1115
	0.1330	0.5449	0.0008 ***	0.0015 **	0.0337 *	0.0056 **	0.0078 **		0.4883	0.7202	0.1166	0.7616
IL-10	−0.4610	−0.7173	−0.1381	−0.5382	−0.6203	−0.3914	−0.3865	−0.2454		0.8997	0.5106	0.5410
	0.1820	0.0240 *	0.7101	0.1117	0.0614 *	0.2599	0.2663	0.4883		0.0009 ***	0.1351	0.1101
IL-6	−0.4377	−0.7212	0.0590	−0.4512	−0.4492	−0.2378	−0.3058	−0.1301	0.8997		0.6000	0.5030
	0.2080	0.0234 *	0.8849	0.1916	0.1929	0.5056	0.3870	0.7202	0.0009 ***		0.0734 *	0.1440
ACRP-30	−0.6128	−0.4667	−0.3999	−0.5671	−0.4101	−0.3476	−0.3853	−0.5326	0.5106	0.6000		0.1394
	0.0643	0.1786	0.2575	0.0923 *	0.2392	0.3231	0.2702	0.1166	0.1351	0.0734 *		0.7072
TNF-α	−0.2376	0.0061	0.2688	−0.2622	−0.1237	0.0000	−0.2080	0.1115	0.5410	0.5030	0.1394	
	0.5065	1.0000	0.4599	0.4613	0.7320	1.0000	0.5619	0.7616	0.1101	0.1440	0.7072	

Statistically significant values are marked with *, as follows: * *p* < 0.1; ** *p* < 0.01; *** *p* < 0.001.

**Table 7:** Correlation data of demographic, clinical characteristics, and biomarkers found in women.

	Age	BMI	Kellgren–Lawrence Score	WOMAC Pain Subscale	WOMAC Stiffness Subscale	WOMAC Physical Function	Total WOMAC Score	VAS Score	IL-10	IL-6	ACRP-30	TNF-α
		−0.4878	0.5359	0.5555	0.6223	0.6098	0.5936	0.4270	0.2121	0.2583	−0.3068	0.1307
Age		0.0787 *	0.0507 *	0.0417 *	0.0203 *	0.0230 *	0.0277 *	0.1286	0.4629	0.3719	0.2856	0.6544
	−0.4878		−0.1960	−0.1747	−0.1473	−0.2483	−0.2159	−0.2281	−0.1116	0.0640	0.1678	−0.3212
BMI	0.0787 *		0.4984	0.5463	0.6129	0.3877	0.4540	0.4285	0.7007	0.8283	0.5637	0.2602
Kellgren–Lawrence	0.5359	−0.1960		0.7862	0.7227	0.8479	0.8342	0.8089	−0.0269	0.2564	0.1848	0.1773
score	0.0507 *	0.4984		0.0011 **	0.0066 **	0.0002 ***	0.0003 ***	0.0007 ***	0.9280	0.3744	0.5251	0.5410
WOMAC pain	0.5555	−0.1747	0.7862		0.8918	0.9665	0.9721	0.8309	−0.3585	−0.0892	0.0736	0.1152
subscale	0.0417 *	0.5463	0.0011 **		0.0001 ***	<0.0001 ****	<0.0001 ****	0.0004 ***	0.2061	0.7612	0.8026	0.6921
WOMAC stiffness	0.6223	−0.1473	0.7227	0.8918		0.8993	0.9066	0.7276	−0.0914	0.0939	0.0868	−0.1142
subscale	0.0203	0.6129	0.0066 **	0.0001 ***		<0.0001 ****	<0.0001 ****	0.0049 **	0.7508	0.7480	0.7668	0.6936
WOMAC physical	0.6098	−0.2483	0.8479	0.9665	0.8993		0.9956	0.8956	−0.2121	0.0177	0.1325	0.2115
function	0.0230 *	0.3877	0.0002 ***	<0.0001 ****	<0.0001 ****		<0.0001 ****	<0.0001 ****	0.4615	0.9544	0.6496	0.4635
Total WOMAC	0.5936	−0.2159	0.8342	0.9721	0.9066	0.9956		0.8957	−0.2419	−0.0265	0.1588	0.2102
score	0.0277 *	0.4540	0.0003 ***	<0.0001 ****	<0.0001 ****	<0.0001 ****		<0.0001 ****	0.3997	0.9304	0.5851	0.4664
VAS	0.4270	−0.2281	0.8089	0.8309	0.7276	0.8956	0.8957		−0.1663	0.0425	0.3423	0.2503
score	0.1286	0.4285	0.0007 ***	0.0004 ***	0.0049 **	<0.0001 ****	<0.0001 ****		0.5651	0.8866	0.2294	0.3840
	0.2121	−0.1116	−0.0269	−0.3585	−0.0914	−0.2121	−0.2419	−0.1663		0.8223	0.1156	−0.0669
IL-10	0.4629	0.7007	0.9280	0.2061	0.7508	0.4615	0.3997	0.5651		0.0006 ***	0.6919	0.8187
	0.2583	0.0640	0.2564	−0.0892	0.0939	0.0177	−0.0265	0.0425	0.8223		0.1560	−0.1963
IL-6	0.3719	0.8283	0.3744	0.7612	0.7480	0.9544	0.9304	0.8866	0.0006 ***		0.5944	0.4983
	−0.3068	0.1678	0.1848	0.0736	0.0868	0.1325	0.1588	0.3423	0.1156	0.1560		−0.0221
ACRP-30	0.2856	0.5637	0.5251	0.8026	0.7668	0.6496	0.5851	0.2294	0.6919	0.5944		0.9425
TNF-α	0.1307	−0.3212	0.1773	0.1152	−0.1142	0.2115	0.2102	0.2503	−0.0669	−0.1963	−0.0221	
	0.6544	0.2602	0.5410	0.6921	0.6936	0.4635	0.4664	0.3840	0.8187	0.4983	0.9425	

Statistically significant values are marked with *, as follows: * *p* < 0.1; ** *p* < 0.01; *** *p* < 0.001; **** *p* < 0.0001.

## Data Availability

Data is contained within the article.
